# Efficient discrimination against RNA-containing primers by human DNA polymerase ε

**DOI:** 10.1038/s41598-022-14602-2

**Published:** 2022-06-17

**Authors:** Alisa E. Lisova, Andrey G. Baranovskiy, Lucia M. Morstadt, Nigar D. Babayeva, Tahir H. Tahirov

**Affiliations:** Eppley Institute for Research in Cancer and Allied Diseases, Fred and Pamela Buffett Cancer Center, University of Nebraska Medical Center, Omaha, NE 68198 USA

**Keywords:** Biochemistry, Structural biology

## Abstract

DNA polymerase ε (Polε) performs bulk synthesis of DNA on the leading strand during genome replication. Polε binds two substrates, a template:primer and dNTP, and catalyzes a covalent attachment of dNMP to the 3' end of the primer. Previous studies have shown that Polε easily inserts and extends ribonucleotides, which may promote mutagenesis and genome instability. In this work, we analyzed the mechanisms of discrimination against RNA-containing primers by human Polε (hPolε), performing binding and kinetic studies at near-physiological salt concentration. Pre-steady-state kinetic studies revealed that hPolε_CD_ extends RNA primers with approximately 3300-fold lower efficiency in comparison to DNA, and addition of one dNMP to the 3′ end of an RNA primer increases activity 36-fold. Likewise, addition of one rNMP to the 3′ end of a DNA primer reduces activity 38-fold. The binding studies conducted in the presence of 0.15 M NaCl revealed that human hPolε_CD_ has low affinity to DNA (*K*_*D*_ of 1.5 µM). Strikingly, a change of salt concentration from 0.1 M to 0.15 M reduces the stability of the hPolε_CD_/DNA complex by 25-fold. Upon template:primer binding, the incoming dNTP and magnesium ions make hPolε discriminative against RNA and chimeric RNA–DNA primers. In summary, our studies revealed that hPolε discrimination against RNA-containing primers is based on the following factors: incoming dNTP, magnesium ions, a steric gate for the primer 2′OH, and the rigid template:primer binding pocket near the catalytic site. In addition, we showed the importance of conducting functional studies at near-physiological salt concentration.

## Introduction

DNA polymerase ε (Polε) is one of the main eukaryotic replicases and is responsible for extension of the leading DNA strand during replication of genetic material^1^. It belongs to the B-family of DNA polymerases, which also includes Polδ, Polα, and Polζ. Human Polε (hPolε) consists of four subunits: the catalytic subunit (p261) and the auxiliary subunits p59, p17, and p12. p261 is a bi-lobe protein comprised of two tandem exonuclease/polymerase domains, the first of which is active, while the second is inactive and plays an essential structural role^2–4^. Two small subunits, p17 and p12, form a heterodimer, which bridges the two lobes of p261^5^. Polε is tethered to the Cdc45-MCM-GINS (CMG) helicase via the N-terminus of p59 and the C-terminal part of p261^4,6,7^.

The accuracy of DNA replication is important for genome stability. Polε achieves the high fidelity of DNA replication due to its accurate polymerase and proofreading exonuclease, which removes incorrect nucleotides from the primer 3′ end. Instability of hPolε holoenzyme drives replication stress, tumorigenesis, and developmental abnormalities^8,9^. Somatic mutations affecting the exonuclease domain of hPolε often occur in hypermutated colorectal and endometrial tumors^10–12^. Many of them cause a mutator or ultra-mutator phenotype upon modelling in yeast system^13–15^.

The vast majority of previous kinetic and DNA binding studies of human and yeast Polε were conducted at low salt concentration or in the absence of it^16–20^, which might affect the interpretation of obtained results. In this work, we performed functional studies of hPolε at near-physiological salt concentration by employing pre-steady-state and binding kinetics. We studied the discrimination of hPolε against RNA-containing primers at template:primer binding and dNTP incorporation steps. In addition, we examined the effect of dNTP, magnesium ions, and salt concentration on the stability of hPolε/template:primer complexes.

## Results

### Salt concentration has a dramatic effect on the stability of the hPolε/DNA complex

**Figure 1:**
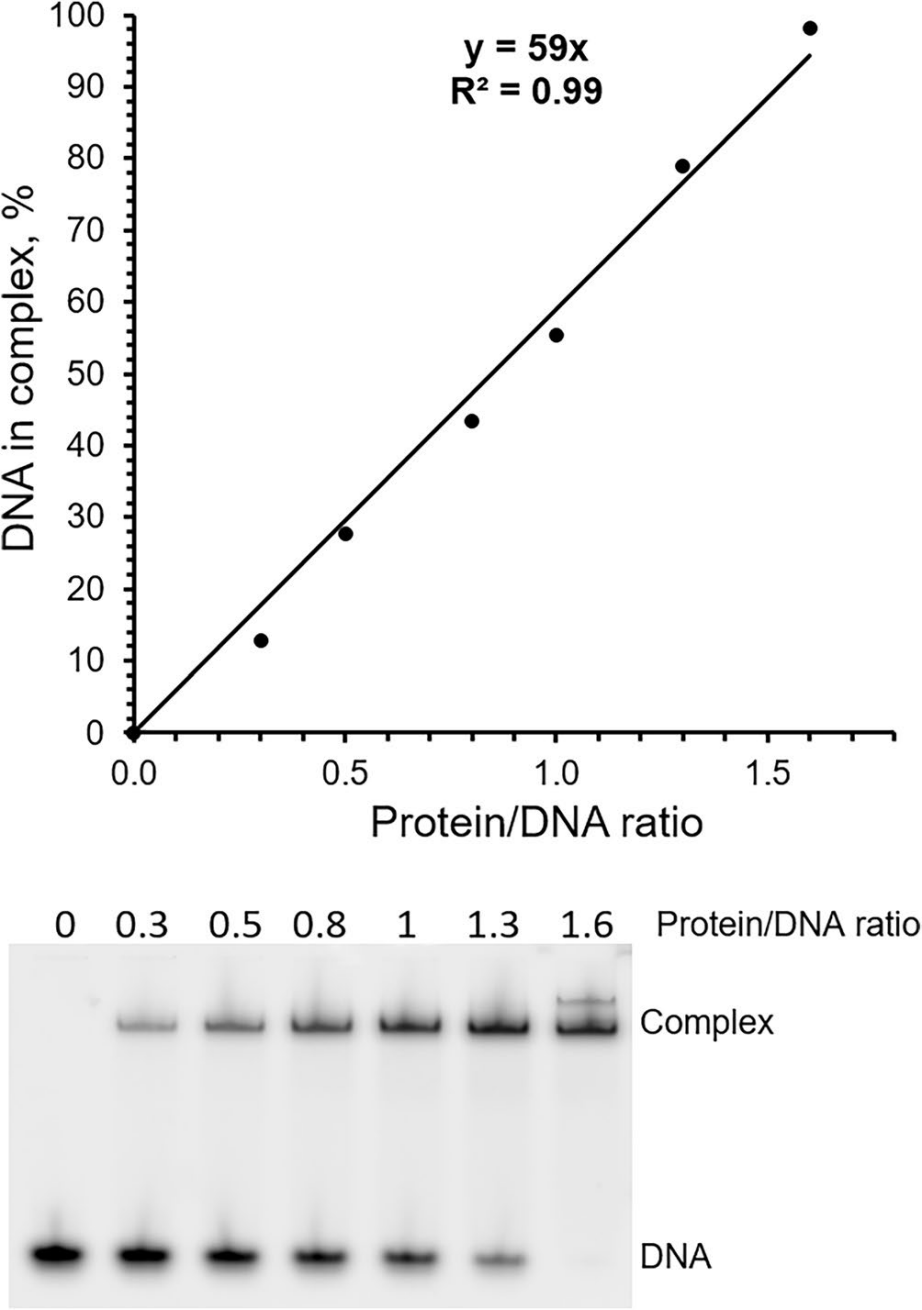
Estimation of the level of active molecules by EMSA. The Cy3-labeled DNA (0.5 µM) was incubated with varying amounts of hPolε_CD_. Samples were separated by electrophoresis in 5% acrylamide gel and visualized by Typhoon FLA 9500. The percentage of DNA in the complex is plotted against the protein/DNA ratio, and the generated trend line shows the percentage of active molecules capable of DNA binding. The result of one representative experiment is shown.

The catalytic domain of human DNA polymerase ε (hPolε_CD_; residues 27-1172) with inactivated exonuclease activity was expressed in *Escherichia coli* and purified to near homogeneity (Suppl. Figure S1). The concentrated sample had a brownish color due to the presence of a [4Fe-4S] cluster^21,22^. Before starting functional studies with the obtained hPolε_CD_ sample, we estimated the level of molecules possessing DNA binding activity. Increasing amounts of protein were added to reactions containing 0.5 µM DNA, and the level of DNA in complex with a protein was estimated by electrophoretic mobility shift assay (EMSA; Fig. 1). It was found that a hPolε_CD_ sample with homogeneity greater than 90% (Suppl. Figure S1) has a level of active molecules of 55.4 ± 4.3%. All data described below were obtained using the active enzyme concentration.

The analysis of Polε_CD_ interaction with DNA (Suppl. Table S1) was performed at near-physiological salt concentration using an Octet K2, which employs Bio-Layer Interferometry (BLI) technology to monitor molecular interactions in real time. This approach allows obtaining the rate constants of complex formation (*k*_on_) and dissociation (*k*_off_) as well as the dissociation constant (*K*_*D*_). A 23-mer DNA template with biotin at the 5′-end was primed by an 11-mer DNA primer and loaded on a streptavidin-coated sensor. Primer length selection was based on structural data showing that ten base-pairs in a duplex is sufficient to form all necessary contacts with the conserved catalytic domain of yeast Polε_CD_^23,24^.

Binding studies revealed that in the presence of 0.15 M NaCl hPolε_CD_ binds a DNA duplex with low affinity (*K*_*D*_ = 1.5 µM, Table 1). The obtained *k*_off_ value of 0.46 s^−1^ indicates that the half-life of the hPolε_CD_/DNA complex is ~ 1.5 s on average. Surprisingly, previous reports showed one to two orders of magnitude stronger interaction with DNA for human and yeast Polε. For example, the reported *K*_*D*_ values for the complexes of hPolε and hPolε_CD_ with DNA are 22 nM and 79 nM, respectively^16,18^. For yeast Polε and Polε_CD_, the corresponding *K*_*D*_ values are 11.6 nM and 15.6 nM, respectively^19^. The discrepancy with our data might be explained by the absence of salt in those studies. Of note, the obtained *k*_on_ value of 304 mM^−1^ s^−1^ (Table 1) is close to the previously reported *k*_on_ value of 270 mM^−1^ s^−1^ acquired by a different approach based on the measurement of DNA polymerase activity^16^.

**Table 1:** Effect of salt concentration and dTTP/Mg^2+^ on interaction of hPolε_CD_ with DNA.

[NaCl] mM	dTTP^a^ Mg^2+^	*k*_off_ × 10^–3^ s^−1^	*k*_on_ (mM^−1^ s^−1^)	*K*_*D*_^b^ (nM)
150	−	463 ± 59	304 ± 20	1519 ± 91
	+	910 ± 29	235 ± 36	3790 ± 321
125	−	138 ± 18	258 ± 7.6	532 ± 53
100	−	21.8 ± 3.9	356 ± 7.2	61.4 ± 12
	+	31.9 ± 5.6	264 ± 24	120 ± 11

Data are presented as mean ± SD (n = 3).

^a^dTTP was added at a concentration of 50 µM, together with 5 mM MgCl_2_.

^b^*K*_d_ values are obtained by dividing *k*_off_ by *k*_on_.

To analyze the effect of salt concentration on the stability of the hPolε_CD_/DNA complex, we also conducted binding experiments in the presence of 0.1 M NaCl. Strikingly, a decrease in salt concentration from 0.15 M to 0.1 M reduced *K*_*D*_ by 25-fold (from 1.52 µM to 61 nM), which is mainly due to the 21-fold reduction in *k*_off_ value (Table 1). Consistently, in the presence of 125 mM NaCl, the hPolε/DNA complex showed a *K*_*D*_ of 532 nM, which is 8.7-fold higher than the *K*_*D*_ value obtained at 100 mM NaCl. The dramatic effect of the ionic strength on the stability of the hPolε_CD_/DNA complex will be discussed below.

We analyzed how the incoming dNTP at near-physiological salt concentration^25^ affects the interaction of hPolε_CD_ with DNA. DNA polymerases use divalent metals to coordinate the triphosphate moiety of dNTP, so magnesium chloride was added to the reaction as well. Addition of 50 µM dTTP and 5 mM MgCl_2_ in the presence of 0.15 M NaCl resulted in a 2.5-fold reduction in affinity of hPolε_CD_ to DNA, with an almost two-fold increase in *k*_off_ value (Table 1). A similar effect was observed at 0.1 M NaCl, with *K*_*D*_ and *k*_off_ being increased by two- and 1.5-fold, respectively. The following exploration revealed that Mg^2+^ ions make a major impact on Polε interaction with a template:primer (see below).

### The incoming dNTP and Mg^2+^ aid hPolε in discrimination against RNA and chimeric primers upon template:primer binding

**Figure 2:**
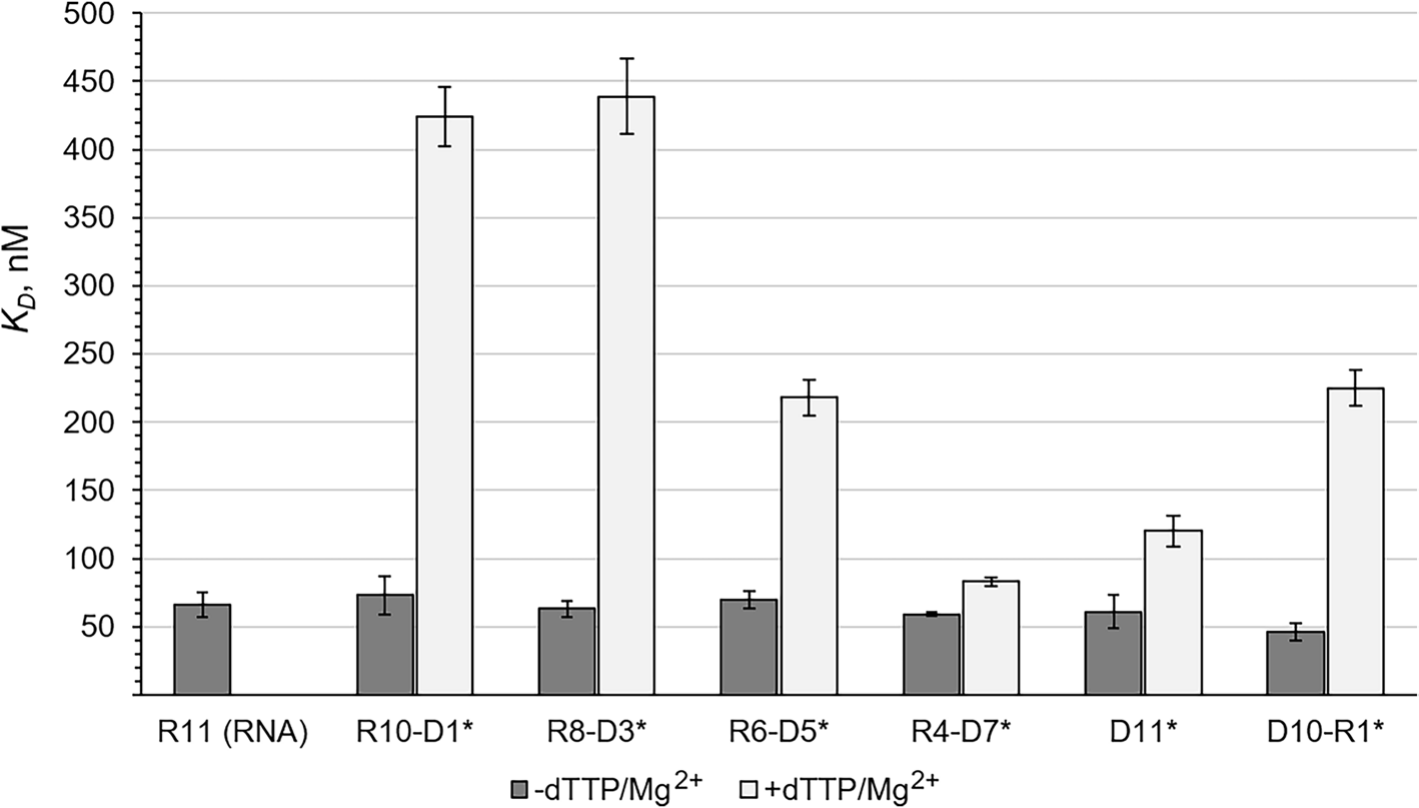
Effect of primer structure and dTTP/Mg^2+^ on hPolε_CD_ affinity to a template:primer. The *K*_*D*_ values from Table 2 are presented as bar graphs showing the mean ± SD (n = 3). The primer type is indicated below the bar. Asterisk indicates the absence of primer 3′-hydroxyl.

We analyzed how hPolε_CD_ discriminates against RNA and chimeric RNA–DNA primers upon binding them in the presence and absence of dTTP/Mg^2+^. Without them, similar *K*_*D*_ values were obtained for all types of primers (Table 2; Fig. 2). The addition of 50 µM dTTP and 5 mM MgCl_2_ reduced the stability of all complexes but to varying extents depending on primer structure. For a chimeric primer with seven dNMPs at the 3′ end, *K*_*D*_ increased only 1.5-fold, which is close to the two-fold increase observed for the DNA primer. In the presence of dTTP/Mg^2+^, the chimeric primers with one or three dNMPs at the 3′ end demonstrate *K*_*D*_ values that are 3.5-fold higher compared to the DNA primer (Table 2; Fig. 2). In comparison, the discrimination factor reduced ~ two-fold for a primer with five dNMPs at the 3′ end.

**Table 2:** Effect of primer structure and dTTP/Mg^2+^ on interaction of human Polε_CD_ with template:primer.

Primer sequence	*k*_off_ × 10^–3^ s^−1^		*k*_on_ (mM^−1^ s^−1^)		*K*_*D*_^a^ (nM)	
	−	+ dTTP	−	+ dTTP	−	+ dTTP
GCCUGGAGCGC (0)	21.9 ± 2.2	–	336 ± 80	–	65.8 ± 8.8	–
GCCUGGAGCG/ddC/ (1)^a^	19.1 ± 2.5	88.6 ± 11	269 ± 38	210 ± 15	73.0 ± 14	424 ± 22
GCCUGGAGCG/ddC/ (3)	19.8 ± 0.1	110 ± 18	318 ± 29	250 ± 24	62.5 ± 6.1	439 ± 28
GCCUGGAGCG/ddC/ (5)	18.2 ± 1.8	58.5 ± 1.7	264 ± 51	269 ± 24	69.8 ± 6.4	218 ± 13
GCCUGGAGCG/ddC/ (7)	15.0 ± 2.1	22.6 ± 2.7	257 ± 31	273 ± 22	58.6 ± 1.3	83 ± 3.3
GCCTGGAGCG/ddC/ (11)	21.8 ± 3.9	31.9 ± 5.6	356 ± 7.2	264 ± 24	61.4 ± 12	120 ± 11
GCCTGGAGCG/3dC/ (10)^b^	17.1 ± 1	54.9 ± 0.2	378 ± 27	244 ± 13	45.6 ± 6.1	225 ± 13

2′-Deoxy- and 2′,3′-dideoxy- nucleotides are underlined and their quantity is indicated in parenthesis.

The provided values for DNA primers at 0.1 M NaCl are from Table 1.

Data are presented as mean ± SD (n = 3).

^a^/ddC/—2′,3′-dideoxy-cytosine.

^b^/3dC/—3′-deoxy-cytosine.

In order to estimate how insertion of one rNMP affects interaction of hPolε with the template:primer, we used a DNA primer with a 3′-deoxy-cytosine. This primer has a 2′-hydroxyl to imitate ribonucleotide and lacks 3′OH to prevent primer extension. Upon addition of dTTP/Mg^2+^, the affinity of hPolε_CD_ to the template:primer decreased five-fold, which is similar to the 5.8-fold reduction observed for an RNA primer with a dideoxy-cytosine at the 3′-end (Table 2; Fig. 2). Direct comparison of affinity to a DNA primer and to a primer with 3′-rNMP demonstrates a 1.9-fold higher selectivity for DNA. Therefore, hPolε senses the primer 2′-hydroxyl only in the presence of incoming dNTP and Mg^2+^.

**Figure 3:**
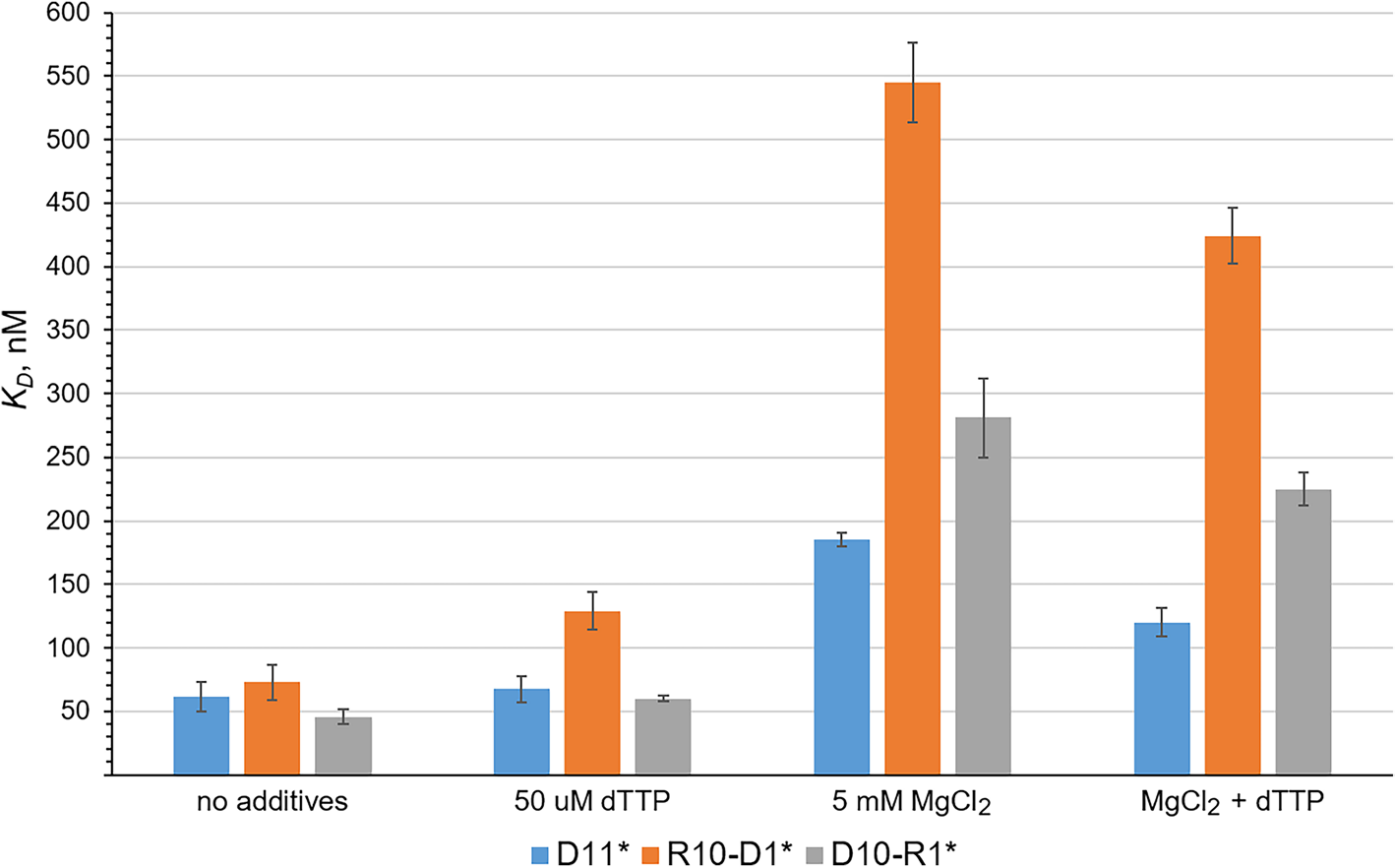
Effect of dTTP and Mg^2+^ on hPolε_CD_ affinity to a template:primer. The data are presented as bar graphs showing the mean ± SD (n = 3). The primer type is indicated below the additives. Asterisk indicates the absence of primer 3′-hydroxyl.

The effect of either 5 mM MgCl_2_ or 50 µM dTTP on hPolε_CD_ affinity to a template:primer was analyzed using the following primers: DNA, R10-D1, and D10-R1 (Fig. 3). Interestingly, these additives added separately or together had a stronger impact on hPolε interaction with RNA-containing primers. The most impressive effect was observed for R10-D1, where addition of either dTTP or Mg^2+^ increased *K*_*D*_ values by 1.8- and 7.5-fold, respectively (Fig. 3). Thus, in the presence of dTTP, hPolε has a 1.9-fold higher affinity to a DNA primer versus R10-D1; in the presence of Mg^2+^, the difference is three-fold. Of note, discrimination against both RNA-containing primers is slightly higher in the presence of dTTP/Mg^2+^ than Mg^2+^ (Fig. 3). These data indicate that, upon ternary complex formation, the incoming dNTP and Mg^2+^ help hPolε to discriminate against RNA-containing primers until they are extended with several dNMPs.

### hPolε extends RNA-containing primers with greatly reduced activity

**Figure 4:**
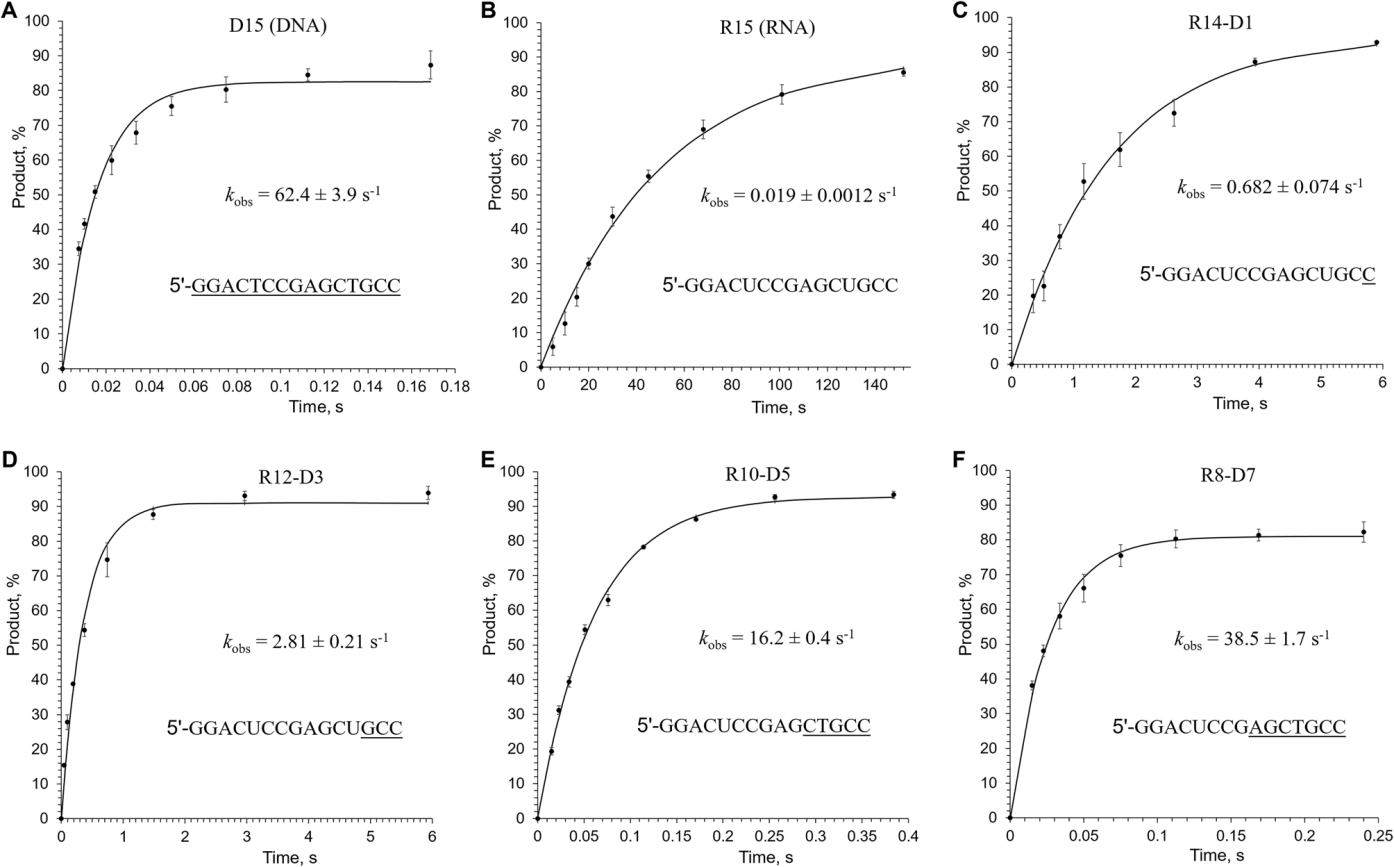
Effect of primer structure on its extension by hPolε_CD_. Activity of hPolε_CD_ in extension of a DNA primer (**A**), an RNA primer (**B**), and chimeric RNA–DNA primers with one (**C**), three (**D**), five (**E**), and seven (**F**) deoxynucleotides at the primer 3′-end was analyzed. Primer sequences are indicated on the graphs; DNA stretches in chimeric primers are underlined. Percentage of extended primer was plotted against time and the data fit to a single-exponential equation (1). Reactions, containing 0.44 µM hPolε_CD_, 0.2 µM template:primer, and 1 mM dTTP, were incubated at 35 °C at the indicated time points. The mean *k*_*obs*_ values and standard deviations were calculated from three independent experiments using GraphPad Prizm software.

It was previously shown that Polε is able to incorporate ribonucleotides into a growing DNA strand and to extend them^26,27^. Moreover, Polε can extend R-loops, which present the DNA template primed by RNA as well as a displaced complementary DNA strand^28^. Accordingly, we decided to explore how the presence of ribonucleotides in a primer affects its extension by hPolε_CD_ by employing the pre-steady-state kinetic approach. Single-nucleotide incorporation experiments were performed under single-turnover conditions by providing a two-fold excess of hPolε_CD_ over DNA (Suppl. Table S1). hPolε_CD_ (0.88 µM) was incubated with a Cy3-labeled DNA (0.4 µM) and quickly mixed with an equal volume of 2 mM dTTP and 10 mM MgCl_2_ under rapid chemical quench conditions. The fraction of extended primer was plotted against time (Fig. 4) and the data were fit to a single-exponential equation (Eq. 1).

hPolε_CD_ extends the DNA primer with a rate of 62.4 s^−1^ obtained at 35 °C in the presence of 0.1 M NaCl (Fig. 4A). In comparison, the catalytic domain of hPolα has a *k*_pol_ of 33.8 s^-1^ at conditions similar to those used in this work^29^. These results are in line with the recently reported average rate of replication fork movement in yeast cells, which is ~ 50 nucleotides per second^30^. A significantly higher *k*_pol_ value of 248 s^-1^ was obtained for the hPolε_CD_ at 20 °C in the absence of salt^16^. The authors of that study noted that the rate of polymerization was > 500 s^−1^ at 37 °C. The almost tenfold difference in activity with our data is probably due to the inhibitory effect of salt concentration as previously demonstrated^31^.

**Figure 5:**
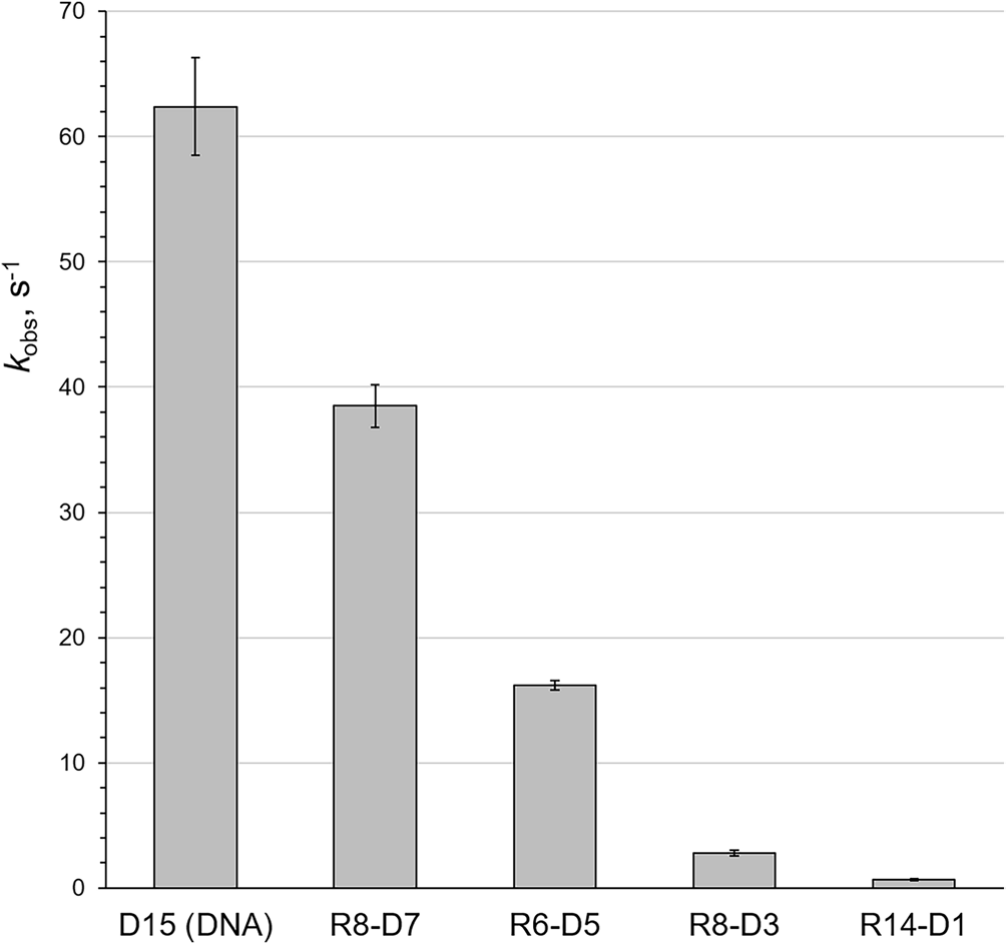
Efficiency of RNA–DNA primer extension by hPolε_CD_ depends on the length of DNA tract at the 3′-end. The *k*_*obs*_ values are presented as bar graphs showing the mean ± SD (n = 3). The type of primer is indicated below the bar.

Activity of hPolε_CD_ in extension of RNA and chimeric primers was analyzed at the same conditions as with the DNA primer. Strikingly, the rate of RNA primer extension is 3300-fold lower compared to DNA (Fig. 4B). Such a severe effect on catalysis indicates that pre-catalytic positioning of the primer 3′-hydroxyl relative to the α-phosphate of dNTP and/or the catalytic metal is changed. Upon addition of dNMPs to the 3′-end of an RNA primer, the rate of primer extension gradually increases (Fig. 4C–F). Thus, the rates of dNMP incorporation on the chimeric RNA–DNA primers with one, three, five, and seven dNMPs at the 3′-end are 92-, 22-, 3.8-, and 1.6-fold lower, respectively, than on DNA (Fig. 5).

**Figure 6:**
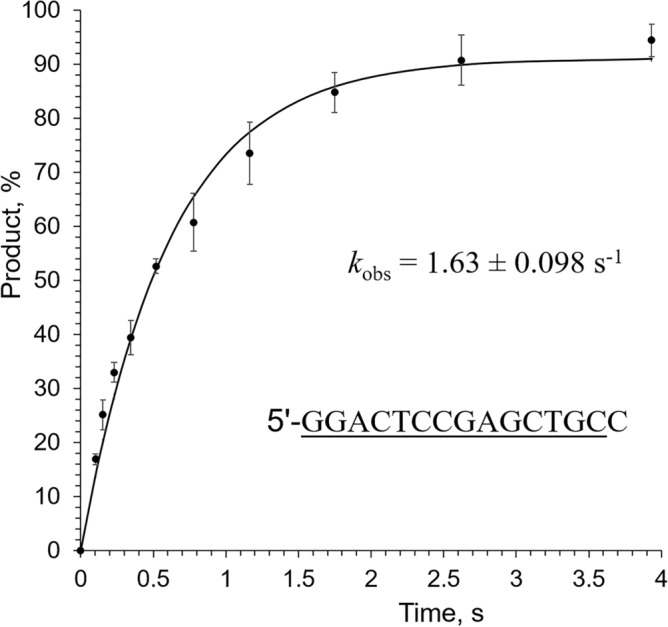
Kinetics of dNMP incorporation into a DNA primer with a ribonucleotide at the 3′-end. Percentage of extended primer was plotted against time and the data fit to a single-exponential equation (Eq. 1). Primer sequence is indicated on the graph with the DNA tract underlined. Reactions, containing 0.44 µM hPolε_CD_, 0.2 µM template:primer, and 1 mM dTTP, were incubated at 35 °C at the indicated time points. The mean *k*_*obs*_ values and standard deviations were calculated from three independent experiments by using GraphPad Prizm software.

**Figure 7:**
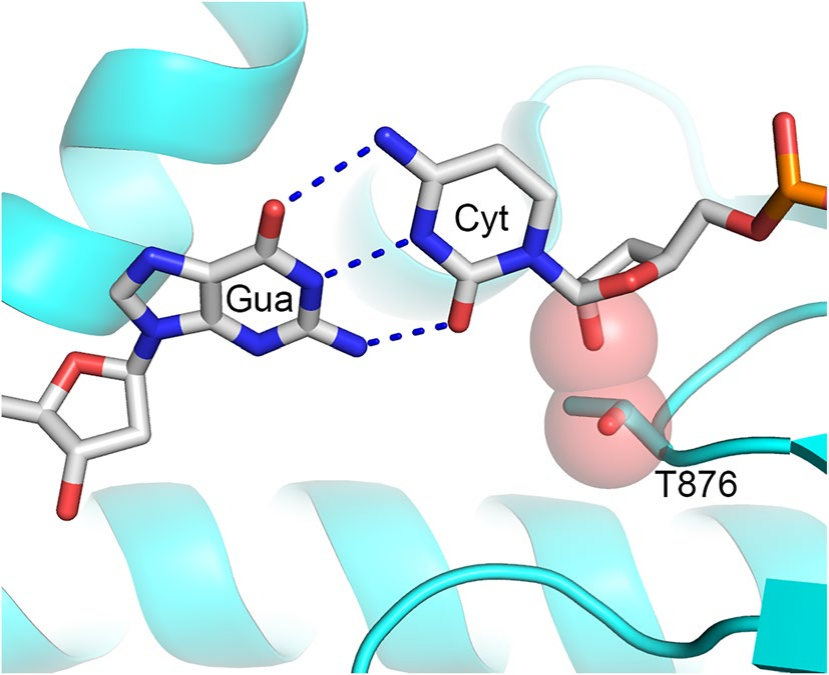
Polε has a steric gate for the 2′-hydroxyl of a primer. The protein is represented as cartoon and colored cyan; Thr876 is shown as sticks. The template and primer are shown as sticks and colored blue for nitrogen, red for oxygen, orange for phosphorus, and grey for carbon. The O1 of Thr876 and modeled O2 of primer Cyt are presented as spheres with 60% transparency and colored red. The interception of spheres indicates steric hindrance. The crystal structure of the ternary complex of yeast Polε_CD_ with DNA and dCTP (pdb code 6hiv (22)) was used for this presentation prepared with the PyMOL Molecular Graphics System.

Interestingly, addition of just one dNMP to the 3′-end of an RNA primer increases activity 36-fold, from 0.019 s^−1^ to 0.682 s^−1^, while the subsequent addition of every two dNMPs increases activity only several-fold (Fig. 4). Moreover, addition of one ribonucleotide to the 3′-end of a DNA primer reduces the rate of dTTP incorporation 38-fold, from 62.4 s^−1^ to 1.63 s^−1^ (Fig. 6). A similar effect on Polε activity upon addition of either 3′-rNMP to a DNA primer or 3′-dNMP to an RNA primer indicates the presence of a steric gate for 2′OH. Indeed, analysis of the structure of yeast Polε_CD_ in complex with DNA and dNTP^23,24^ revealed a conserved Thr876 (Suppl. Figure S2) located under the sugar and sensing the 2′-hydroxyl of a primer (Fig. 7). The other steric gate is reserved for preventing rNTPs incorporation, where a conserved tyrosine residue clashes with a 2′-hydroxyl of an incoming nucleotide triphosphate^32^.

## Discussion

Protein-DNA interactions are mainly electrostatic in nature, which makes them sensitive to ionic strength. If the inhibitory effect of salt is due to competition of ions with charged groups of Polε and DNA involved in interaction, it should be proportional to the salt concentration. Despite this prediction, we have shown that salt severely affects the interaction of hPolε_CD_ with DNA. In particular, a 1.5-fold increase in salt concentration from 0.1 M to 0.15 M results in a 25-fold reduction in the stability of the hPolε_CD_/DNA complex (Table 1). Such a dramatic effect of salt concentration on affinity to DNA can be attributed to changes in protein solvation and elevated conformational dynamics of the flexible parts/domains. The catalytic domains of B-family DNA polymerases have the universal “right-hand” DNA polymerase fold^33^ consisting of five subdomains: N-terminal, exonuclease, palm, fingers, and thumb. Among them, the fingers are the most flexible and change the conformation from “open” to “closed” upon dNTP binding. The thumb domain grips the distant part of the DNA duplex and demonstrates significant flexibility according to structural studies of hPolα^29^. An ordered movement of the palm domain during polymerase translocation along the duplex was proposed from structural studies of yeast Polα^34^.

This study has shown that hPolε_CD_ exhibits low affinity to the template:primer at physiological salt concentration, with a 19-fold higher *K*_*D*_ value than the 79 nM obtained in the absence of salt^16^. Interestingly, hPolα shows a similar with Polε affinity to DNA in the presence of 100 mM (*K*_*D*_ = 41 nM) and 150 mM NaCl (*K*_*D*_ = 1.4 µM), despite the absence of the so-called processivity domain that is unique to Polε^35^. According to structural data^22–24^, this domain makes three to four additional hydrogen bonds with a template:primer (a detailed comparison of hPolα and yPolε interactions with a template:primer are provided in^36^). In other work, we have shown that hPolε_CD_ with an intact exonuclease active site and without internal truncations also exhibits a strong sensitivity to ionic strength upon template:primer binding^37^.

The results of our binding studies support the idea that PCNA is required for processive synthesis of the leading strand, according to the current model of the eukaryotic replication fork^38^. The recently published structure of yeast Polε holoenzyme revealed that accessory subunits do not interact with the DNA duplex^5^, indicating that the holoenzyme footprint on a DNA duplex is only ten base-pairs, the same as for the catalytic domain^22,24^. Polε is flexibly attached to the CMG helicase through interaction with the C-terminal domain of the catalytic subunit and the N-terminal domain of the second accessory subunit^4,7^. In contrast to PCNA, interaction with CMG does not prevent the Polε catalytic domain from occasional dissociation from the growing primer end.

The main role of Polε is a synthesis of the leading DNA strand, but it may handle RNA and chimeric RNA–DNA strands in certain circumstances. One example is a ribonucleotide at the growing primer end, which Polε can insert and extend^26,27^. Another example is the ability of Polε to extend the 3′-ends of R-loops, serving as a possible way to restart DNA replication^28^. In addition, it is possible that Polα sometimes preliminarily terminates DNA synthesis due to generation of a mismatch, like purine-purine, which is difficult to extend. In this case, Polε may bind the chimeric RNA–DNA primer, proofread the non-cognate nucleotide, and extend the corrected primer with dNMPs until the length of a primer is enough for RFC to load PCNA. A similar mechanism was suggested for the lagging strand where Polδ proofreads the Polα-generated mismatches^39^. Intriguingly, some portion of hPolε is associated with RNA polymerase II independently of the cell cycle^40^. Moreover, according to results of UV cross-linking studies, hPolε is located in close proximity to the newly synthesized RNA strand^40^. These data suggest that Polε could play a currently unknown role in RNA transcript processing.

As we found, hPolε_CD_ shows approximately 3,000-fold lower activity in extension of RNA versus DNA primers, making it unlikely that Polε plays a role in restarting the replication fork from the R-loops. The *k*_*obs*_ value of 0.019 s^−1^ indicates that half of RNA primers will be extended with one dNMP in ~ 40 s. The best candidate for R-loop extension would be Polα, which displays a similar rate of DNA and RNA primer extension^29^. On the other hand, upon extension of an RNA primer with dNMPs, hPolε_CD_ activity gradually increases, and the chimeric primer with seven dNMPs is extended fairly well (Fig. 4). Our studies have shown that, in comparison to template:primer binding, the rate of dNMP incorporation is more sensitive to the presence of ribonucleotides in the primer. It is interesting that despite their similarly organized DNA binding sites^29^, Polα and Polε demonstrate such a significant difference in selectivity to a DNA primer upon catalysis of phosphodiester bond formation.

Incorporation of ribonucleotides to the nascent DNA strand by replicative DNA polymerases could be a challenge for genome integrity^41,42^. It was reported previously that hPolε_CD_ readily inserts ribonucleotides, and almost half of them escape proofreading by intrinsic exonuclease^27^. Interestingly, activity of hPolε was reduced only two- to three-fold when one–three consecutive ribonucleotides were added to the primer 3′-end^27^. Such a low effect of 3′-rNMPs on primer extension may be due to the absence of salt in reaction and to the type of assay where the rate of enzyme/DNA complex formation limits the reaction rate due to low concentrations of DNA and Polε and the presence of 10% glycerol. Our study, performed at single-turnover conditions and in the presence of 0.1 M NaCl, demonstrates much stronger sugar selectivity, with a 38-fold reduction in activity by 3′-rNMP (Fig. 6). Due to the balance between DNA polymerase and exonuclease activities^43^, the hampered extension of 3′-ribonucleotides will result in increased probability of their excision.

A notable finding of the current study is the role of incoming dNTP and magnesium ions in the discrimination of hPolε against primers containing ribonucleotides. When dNTP and Mg^2+^ are not present, hPolε binds DNA, RNA, and chimeric primers with similar affinity. In the presence of dTTP/Mg^2+^, selectivity for DNA primers increases two- to four-fold. Interestingly, Mg^2+^ ions play the main role in discrimination against RNA-containing primers upon template:primer binding (Fig. 3). Magnesium ions most likely interact with DNA phosphates and compete with DNA-binding residues of hPolε_CD_. In the case of RNA-containing primers, a stronger effect of Mg^2+^ on the hPolε/template:primer interaction can be due to steric hindrances generated by the wide helix of DNA:RNA duplexes and by the primer 2′-hydroxyl. Of note, dTTP alone has a stronger impact on hPolε affinity to the R10-D1 primer than to DNA (Fig. 3). This is an interesting example of how one substrate modulates enzyme selectivity for another. Noteworthy, there is no discrimination against RNA primers extended with seven or more dNMPs.

Available structural data provide clues as to why, upon template:primer binding, hPolε discriminates against the chimeric primers with one or three dNMPs with similar efficiency and does not discriminate against primers containing seven dNMPs at the 3′ end. Polε interacts with a ten base-pair duplex, where the first four base pairs from the growing primer end interact with the rigid DNA-binding pocket established mainly by the palm subdomain with significant contributions by the main-chain atoms^22–24^. The remainder of the template:primer interacts with the flexible thumb, which can adjust its conformation to accommodate a wider DNA:RNA duplex. The absence of discrimination against RNA when dNTP and Mg^2+^ are not added may be due to the slightly different mode of duplex binding, which reduces steric hindrance between the wide DNA:RNA duplex and the rigid DNA-binding cleft. dNTP and Mg^2+^ binding and the fingers closing likely forces hPolε interaction with the template:primer in a specific way, which results in steric hindrance and reduced affinity to RNA and chimeric primers.

## Materials and methods

### Cloning, expression, and purification

The catalytic domain of hPolε (hPolε_CD_), with the proofreading 3′ → 5′ exonuclease inactivated by the double mutation D275N/E277Q, was cloned into a pASHSUL-1 plasmid^44^ to produce the recombinant protein with a cleavable N-terminal His_6_-Sumo tag. To increase protein solubility and yield, we deleted the external loop spanning amino acids 185–209. The corresponding region is distant from the active center and is mostly disordered in all yeast Polε_CD_ structures.

hPolε_CD_ were expressed in *E. coli* strain Rosetta-2 (DE3) at 18 °C for 16 h following induction with 0.2 µg/ml anhydrotetracycline. Afterward, the cells were harvested by centrifugation at 4000 g for 15 min, washed with PBS, aliquoted and maintained at − 80 °C.

The purification protocol includes chromatography on a Ni-IDA column (Bio-Rad), His-Sumo-tag digestion during overnight dialysis, and chromatography on a Heparin HP HiTrap column (GE Healthcare) as well as on a size-exclusion column in buffer containing 25 mM Tris-HEPES (pH 7.8), 0.15 M NaCl, 1% glycerol, and 2 mM tris(2-carboxyethyl)phosphine (TCEP). Finally, samples were concentrated to 30–60 µM and flash-frozen in aliquots. Protein concentrations were estimated by measuring the absorbance at 280 nm and using extinction coefficients of 154 mM^−1^ cm^−1^. The extinction coefficients were calculated with ProtParam^45^.

#### Estimation of the level of DNA-binding (active) molecules by electrophoretic mobility shift assay

Reactions containing 0.5 µM DNA (Suppl. Table S1) and varying amounts of protein were incubated in 10 μl for 5 min at room temperature in the buffer containing 20 mM Tris-Hepes (pH 7.8), 100 mM NaCl, 2% glycerol, 2 mM TCEP, and 0.2 mg/mL BSA; 5 μl was then loaded on 5% native PAGE. Samples labeled with Cy3-dye were visualized using the Typhoon 9410 imager (GE Healthcare) and quantified using ImageJ software (version 1.53 s, National Institutes of Health). The mean value and standard deviation are calculated from three independent experiments.

#### Binding studies

Analysis of binding kinetics was done at 23 °C on an Octet K2 (Sartorius AG). This device uses Bio-Layer Interferometry technology to monitor molecular interactions in real time. A template with a biotin-TEG at the 5′-end was annealed to the primers (Suppl. Table S1) and immobilized on a streptavidin-coated biosensor (SAX, Sartorius AG). Primers were added at two-fold molar excess over the template. SAX sensors were loaded with oligonucleotide-biotin at 50 nM concentration for 7 min at 500 rpm. Then sensors were blocked by incubating for 2 min in 10 µg/ml biocytin. In the first row of a 96-well microplate (Greiner Bio-One), the first six wells contained the buffer, consisting of 30 mM Tris-Hepes, pH 7.8, 100 mM or 150 mM NaCl, 2 mM TCEP, and 0.002% Tween 20. The next six wells contained two-fold dilutions of hPolε_CD_ in the same buffer. All wells in the adjacent row contained only the buffer for reference. Examples of binding curves are shown in Suppl. Figure S3, where the effect of either 5 mM MgCl_2_ or 50 µM dTTP on the stability of the hPolε_CD_/DNA complex was analyzed. Data Analysis HT software (ver. 11.1, Sartorius AG) was used to calculate binding constants (*k*_on_, *k*_off_, and *K*_*D*_) by using global fitting. The average value and standard deviation were calculated from three independent experiments.

#### Kinetic studies

Pre-steady-state kinetic studies were performed on the QFM-4000 rapid chemical quench apparatus (BioLogic, France) at 35 °C. Reactions contained 0.44 µM hPolε_CD_ (active molecules), 0.2 µM DNA, 1 mM dTTP, 25 mM Tris-HEPES, pH 7.8, 0.1 M NaCl, 5 mM MgCl_2_, 2 mM TCEP, and 0.2 mg/mL BSA. hPolε_CD_ was incubated with a Cy3-labeled 15-mer primer annealed to a 25-mer DNA template (Suppl. Table S1), to allow formation of the binary complex, and rapidly mixed with a solution containing 2 mM dTTP and 10 mM MgCl_2_ followed by quenching with 0.3 M EDTA. Products were collected in a tube containing 15 µl 100% formamide and separated by 20% Urea-PAGE. The Cy3-labeled products were visualized using the Typhoon FLA 9500 (GE Healthcare) and quantified by ImageJ, version 1.53 (NIH). The extended primer fraction was calculated by dividing the amount of extended primer by the amount of primer added in reaction. The percent of extended primer was plotted against time and the data fit to a single-exponential equation:

1 $$  \left[ {{\text{Product}}} \right]{\mkern 1mu}  = A \times {\mkern 1mu} \left( {1 - e^{{ - k_{{{\text{obs}}}} t}} } \right) $$

where *A* is the amplitude, *k*_*obs*_ is the observed rate for dNTP incorporation, and *t* is the time.

## Supplementary Information

Supplementary Information.

## Data Availability

The data that support the findings of this study are included in the Supplementary Information file or available from the corresponding author on request.

## Abbreviations

hPolε: Human DNA polymerase ε
hPolε_CD_: Catalytic domain of human DNA polymerase ε
CMG: Cdc45-MCM-GINS
EMSA: Electrophoretic mobility shift assay
TCEP: Tris(2-carboxyethyl)phosphine
