# Supplementary material for: Efficient discrimination against RNA-containing primers by human DNA polymerase ε
Source: Sci Rep. 2022 Jun 17;12:10163. doi: 10.1038/s41598-022-14602-2 (PMC9205888; doi:10.1038/s41598-022-14602-2)

SUPPLEMENTAL INFORMATION

for the article

Efficient discrimination against RNA-containing primers by human DNA polymerase ε

Alisa E. Lisova, Andrey G. Baranovskiy, Lucia M. Morstadt, Nigar D. Babayeva, Tahir H. Tahirov

Tahir H. Tahirov
Email: ttahirov@unmc.edu

Table S1. Oligonucleotides used in this study

Sequence	Description	Application	Length
5'-AATGTTTCTA*GGCAGCTCGGAGTCC* [a]	Template (DNA)		25
5'-/Cy3/GGACTCCGAGCTGCC (D15)	Primer (DNA)		15
5'-/Cy3/GGACTCCGAGCTGCC [b] (D14-R1)	Primer (chimeric)		15
5'-/Cy3/GGACUCCGAGCUGCC (R15)	Primer (RNA)	kinetic studies	15
5'-/Cy3/GGACUCCGAGCUGCC (R14-D1)	Primer (chimeric)		15
5'-/Cy3/GGACUCCGAGCUGCC (R12-D3)	Primer (chimeric)		15
5'-/Cy3/GGACUCCGAGCTGCC (R10-D5)	Primer (chimeric)		15
5'-/Cy3/GGACUCCGAGCTGCC (R8-D7)	Primer (chimeric)		15
5'-/BiotinTEG/AATACATAA*GCGCTCCAGGC*AAT	Template (DNA)		23
5'-GCCUGGAGCGC (R11)	Primer (RNA)		11
5'-GCCUGGAGCG/ddC/ (R10-D1)	Primer (chimeric)		11
5'-GCCUGGAGCG/ddC/ (R8-D3)	Primer (chimeric)		11
5'-GCCUGGAGCG/ddC/ (R6-D5)	Primer (chimeric	Octet K2	11
5'-GCCUGGAGCG/ddC/ (R4-D7)	Primer (chimeric)		11
5'-GCCTGGAGCG/3ddC/ (D11)	Primer (DNA)		11
5'-GCCTGGAGCG/3dC/ (D10-R1)	Primer (chimeric)		11
5'-ATTAT*GGCAGCTCGGAGTCC*	Template (DNA)	EMSA	20
5'-/Cy3/GGACTCCGAGCTGCC (D15)	Primer (DNA)		15

[a] The template regions complementary to a primer are in italics.
[b] Deoxy- and dideoxy- nucleotides in the chimeric primer sequences are underlined.

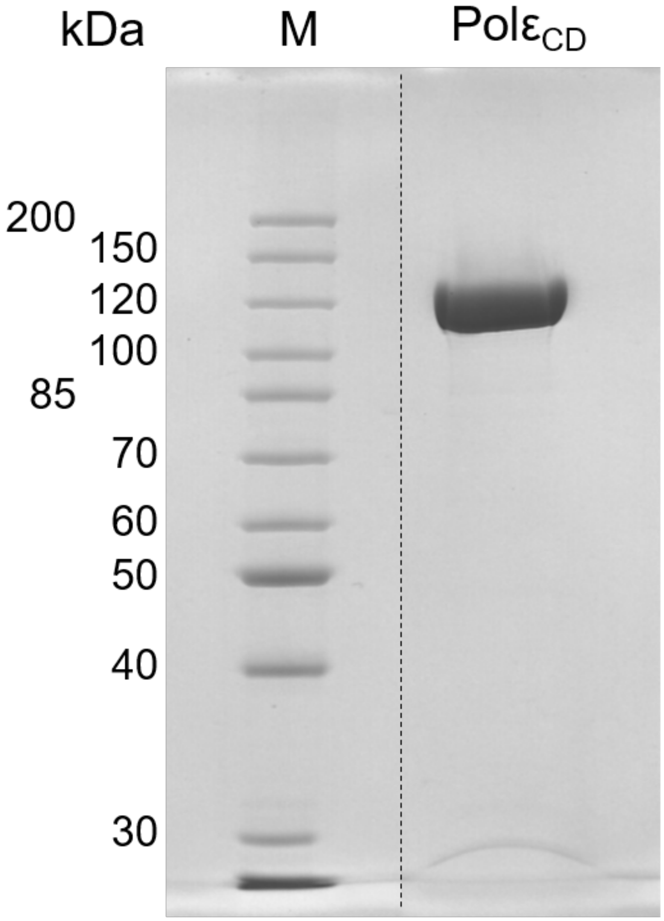

Figure S1. Analysis of hPolε$_{CD}$ purity. Proteins were separated by 8% SDS-PAGE and stained by Coomassie Brilliant Blue R-250. M – molecular weight markers. The dashed line indicates splicing of the original image.

```
Homo sapiens              832 AGIVCFTGANIITQARELIEQIGRPLELDTDGIWCVLPNSFPENFVFKTTNVKK 885
Mus musculus              832 AGIVCFTGANIITQARELIEQIGRPLELDTDGIWCVLPNSFPENFVIKTTNAKK 885
Drosophila melanogaster   830 AGIVCLTGSNIITKAREIIERVGRPLELDTDGIWCILPGSFPQEFTIHTSHEKK 883
Arabidopsis thaliana      805 AGVVTYTGAKIIQNARLLIERIGKPLELDTDGIWCCLPGSFPENFTFKTIDMKK 858
Aspergillus nidulans      816 AGVTCLTGARIIQMARELVERIGRPLELDTDGIWCMLPGTFPENFSFTLKNGKK 869
Saccharomyces cerevisiae  847 AGITCLTGATIIQMARALVERVGRPLELDTDGIWCILPKSFPETYFFTLENGKK 900
```

Figure S2. Thr861 of hPolε is conserved. Amino acid sequence alignment was performed using ClustalW. The conserved and similar residues are highlighted with red and yellow, respectively. The conserved threonines are boxed.

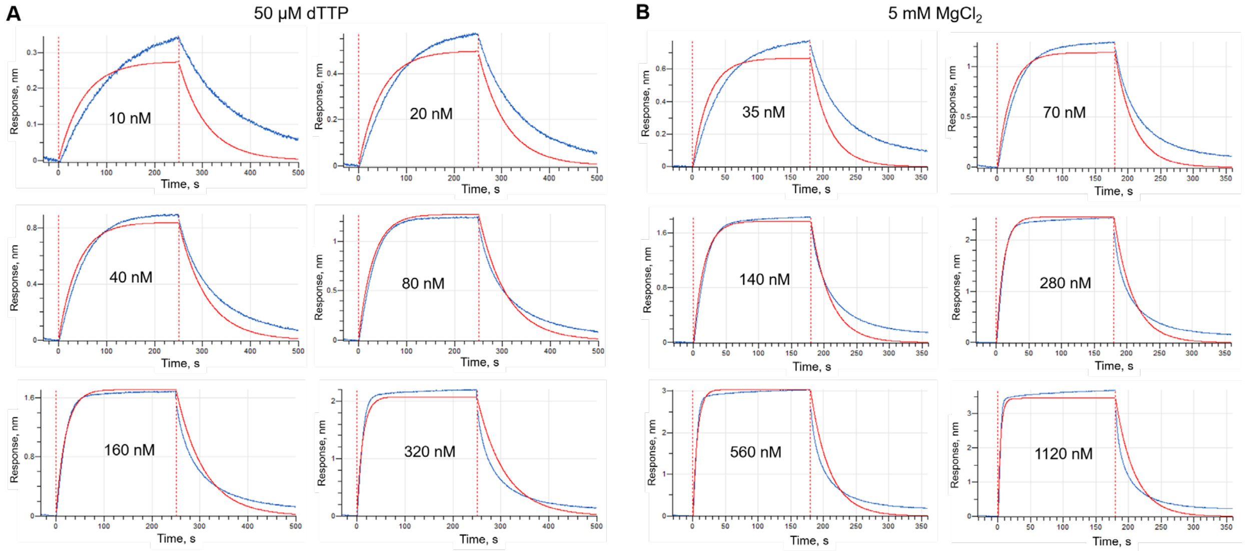

Figure S3. Kinetics of hPolε$_{CD}$/DNA complex formation and dissociation in the presence of 50 µM dTTP (*A*) or 5 mM MgCl$_2$ (*B*). Binding studies were conducted in the buffer containing 30 mM Tris-Hepes, pH 7.8, 100 mM NaCl, 2 mM TCEP, and 0.002% Tween 20. Enzyme concentration is indicated on each graph. DNA is attached to the streptavidin coated biosensor (SAX, Sartorius AG). Each binding cycle consists of next steps: baseline, association, and dissociation. The blue line depicts the actual binding curve. The red line represents the model curve build by the Data Analysis HT software (Sartorius AG) with use of global fitting.

Fig. 1.

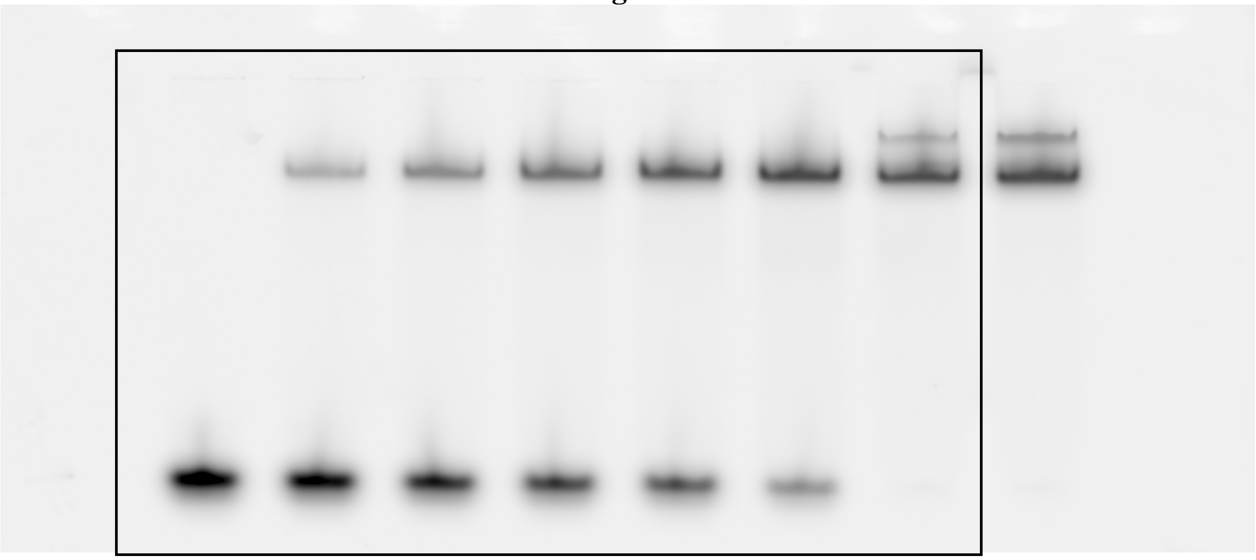

Supp. Fig. S1.

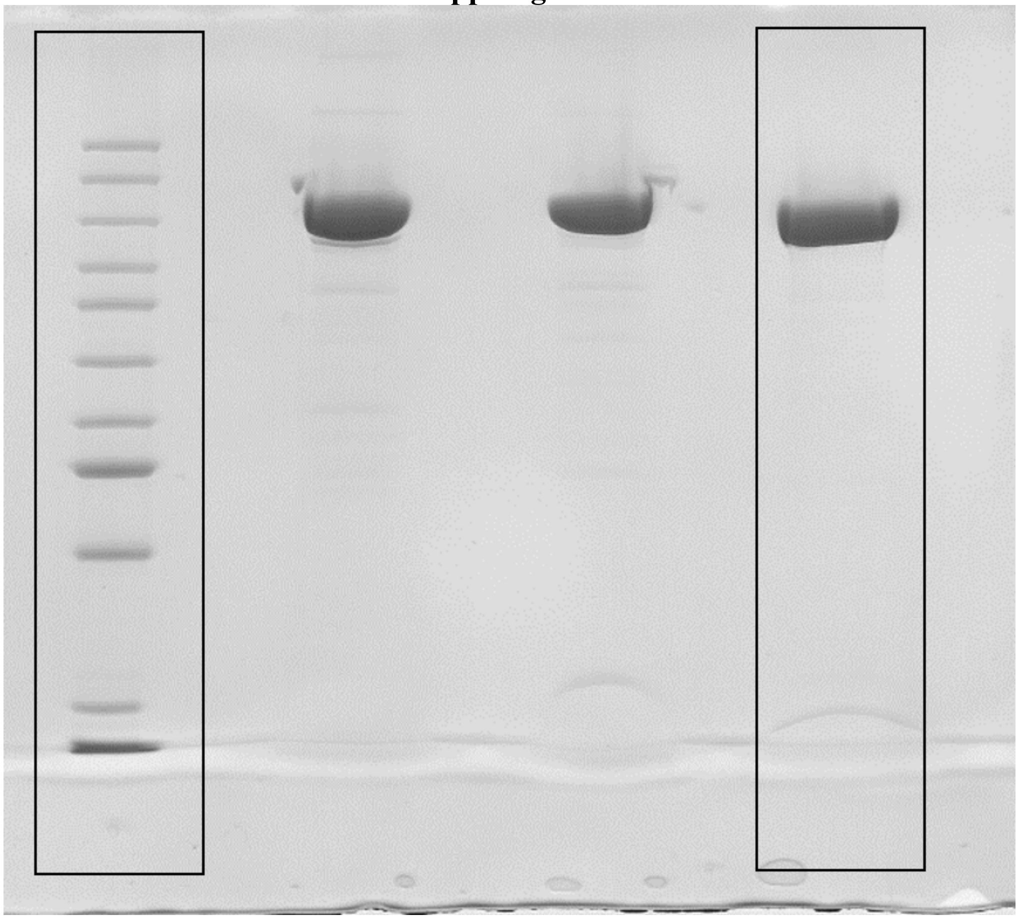

Supplement: Supplementary file 1 — Supplementary Information. [file 41598_2022_14602_MOESM1_ESM.pdf]
